# Amino Acid Region 1000–1008 of Factor V Is a Dynamic Regulator for the Emergence of Procoagulant Activity*

**DOI:** 10.1074/jbc.M113.462374

**Published:** 2013-10-31

**Authors:** Joesph R. Wiencek, Mahesheema Na, Jamila Hirbawi, Michael Kalafatis

**Affiliations:** From the ‡ Department of Chemistry, Cleveland State University, Cleveland, Ohio 44115,; the § Department of Molecular Cardiology, Lerner Research Institute, Cleveland Clinic, Cleveland, Ohio 44195,; the ¶ Center for Gene Regulation in Health and Disease, Cleveland State University, Cleveland, Ohio 44115, and; the ‖ Taussig Cancer Center, Cleveland Clinic, Cleveland, Ohio 44195

**Keywords:** Coagulation Factors, Phospholipid Vesicle, Protein Chemistry, Prothrombin, Thrombin, Factor V, Factor Va, Factor Xa, Prothrombinase, Prothrombin

## Abstract

Single chain factor V (fV) circulates as an *M*_r_ 330,000 quiescent pro-cofactor. Removal of the B domain and generation of factor Va (fVa) are vital for procoagulant activity. We investigated the role of the basic amino acid region 1000–1008 within the B domain of fV by constructing a recombinant mutant fV molecule with all activation cleavage sites (Arg^709^/Arg^1018^/Arg^1545^) mutated to glutamine (fV^Q3^), a mutant fV molecule with region 1000–1008 deleted (fV^ΔB9^), and a mutant fV molecule containing the same deletion with activation cleavage sites changed to glutamine (fV^ΔB9/Q3^). The recombinant molecules along with wild type fV (fV^WT^) were transiently expressed in COS-7L cells, purified, and assessed for their ability to bind factor Xa (fXa) prior to and following incubation with thrombin. The data showed that fV^Q3^ was severely impaired in its interaction with fXa before and after incubation with thrombin. In contrast, *K_D_*_(app)_ values for fV^ΔB9^ (0.9 nm), fVa^ΔB9^ (0.4 nm), and fV^ΔB9/Q3^ (0.7 nm) were similar to the affinity of fVa^WT^ for fXa (0.3 nm). Two-stage clotting assays revealed that although fV^Q3^ was deficient in its clotting activity, fV^ΔB9/Q3^ had clotting activity comparable with fVa^WT^. The *k*_cat_ value of prothrombinase assembled with fV^ΔB9/Q3^ was minimally affected, whereas the *K_m_* value of the reaction was increased 57-fold compared with the *K_m_* value obtained with prothrombinase assembled with fVa^WT^. These findings strongly suggest that amino acid region 1000–1008 of fV is a regulatory sequence protecting the organisms from spontaneous binding to fXa and unnecessary prothrombinase complex formation, which in turn results in catastrophic physiological consequences.

## Teaser

**Background:** Factor V is activated to factor Va to interact with factor Xa.

**Results:** Elimination of nine amino acids from the B domain results in binding of unactivated factor V to factor Xa.

**Conclusion:** Amino acids 1000–1008 of factor V prevent unwanted prothrombinase assembly.

**Significance:** A short peptide sequence from the B region is a regulatory domain for the generation of factor Va procoagulant activity.

## Introduction

**FIGURE 1.**
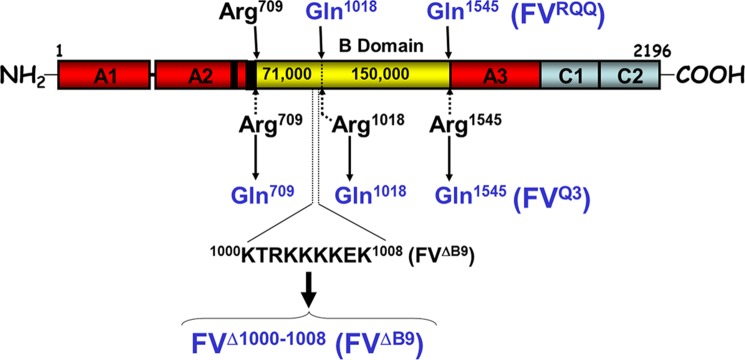
**FV structure and mutant molecules.** The pro-cofactor fV is composed of three A domains (*red*), a connecting B domain (*yellow*), and two C domains (*blue*). fV undergoes three sequential cleavages by thrombin at Arg^709^, Arg^1018^, and Arg^1545^ to generate the active cofactor fVa. The deletion within the basic homologous region of the B domain (amino acid residues 1000–1008) is coupled with mutations at each one of the thrombin activation sites (Arg → Gln). The recombinant mutant fV molecules created are indicated with specific designations used throughout this work.

Human factor V (fV)^3^ circulates in whole blood as a single chain inactive pro-cofactor with an *M*_r_ 330,000 consisting of multiple domains (A1-A2-B-A3-C1-C2) at a concentration of 20 nm (1). In the presence of a procoagulant membrane surface, fV undergoes limited proteolysis to become an active participant in the coagulation cascade (1–3). Single chain fV does not bind fXa, and proper removal of the B domain is vital to generate procoagulant activity (4, 5). fV is activated to factor Va (fVa) by thrombin, fXa, and a protease extracted from a snake venom (RVV-V activator) (1, 2). The sequential cleavage of fV by thrombin at Arg^709^, Arg^1018^, and Arg^1545^ is physiologically necessary for generation of procoagulant activity (Fig. 1) (6). Following the removal of the heavily glycosylated B domain, the light (*M*_r_ 74,000) and heavy (*M*_r_ 105,000) chains of fVa associate in the presence of divalent metal ions into a noncovalently attached heterodimer that functions as a cofactor in the prothrombinase complex (1, 7–9). fVa binds to fXa on a phospholipid membrane in the presence of divalent metal ions to form the prothrombinase complex. Appropriate binding of fVa to fXa during prothrombinase assembly and function is essential for the proper and timely activation of the substrate prothrombin (5, 10, 11).

**FIGURE 2.**
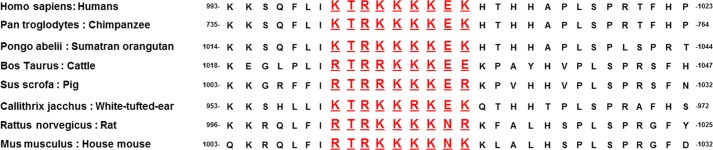
**Comparison of the basic region of amino acid sequences 1000–1008 from fV B domain among multiple species.** The databases GenBank^TM^ and NCBI Trace Archive were used to derive sequences of fV among various mammalian species to compare homology. The basic amino acid sequence of interest is shown in *red*. The following species are included (from *top* to *bottom*): *Homo sapiens*, human; *Pan troglodytes*, chimpanzee; *Pongo abelii*, Sumatran orangutan; *Bos taurus*, cattle; *Sus scrofa*, pig; *Callithrix jacchus*, white-tufted-ear marmoset; *Rattus norvegicus*, Norway rat; *Mus musculus*, western European house mouse.

Removal of a considerable quantity of amino acids from fV to produce an active cofactor molecule has been a major area of study in the expression of recombinant fVa coagulant activity (12–15). Partial to full B domainless fV derivatives have been used to determine the purpose of the B domain and how it participates in the regulation of the coagulation cascade. Kane *et al.* (13) used a recombinant B domainless fV derivative that was missing 680 amino acids (factor V(des-811–1491) or FV-810). FV(des-811–1491) was able to express full coagulant activity after proteolysis with RVV-V or thrombin. Similar results were obtained with other recombinant fV derivatives that were missing more than 50% of the B domain (15). With the introduction of 378 or more amino acids back into this region, other mutants became functionally deficient in clotting activity and were comparable with the pro-cofactor molecule (15). However, one recombinant molecule, factor V-956, which was constructed without a specific region rich in basic amino acids, had an intermediate activity. In the portion 963–1008, 18 of 46 amino acids were identified to be either lysine or arginine, and 7 of these 18 amino acids are located in a row within the region encompassing amino acids 1000–1008 (Fig. 2) (15).

Although the B domain of fV does not share homology with factor VIII or other known proteins, a comparative sequence analysis of fV among mammals revealed that a stretch of amino acids within a highly basic region was consistently found in other species attesting to its physiological significance (Fig. 2). Thus, the basic amino acid sequence 1000–1008 of the B domain of fV could play a part in inhibiting binding between fXa and the heavy and/or light chain of fVa (Fig. 1) (16–21). It is noteworthy that we and others have demonstrated that the binding site for fXa located on the heavy chain of the cofactor is composed of a majority of acidic amino acids (17–22). Proteolysis of fV and removal of this basic amino acid region may allow for the release of electrostatic restraints and the ability for unactivated fV to show procoagulant activity (*i.e.* interact with fXa on a procoagulant membrane surface and activate prothrombin). Thus, this mechanism that controls the pro-cofactor would be comparable with certain aspartic and cysteine proteases (23).

This work was undertaken to explore the significance of the basic amino acid sequence that is almost identical among a wide range of species (Fig. 2) and to understand its role in maintaining fV in a quiescent state. The aim of our study was to verify the hypothesis that amino acid region 1000–1008 provides a specific regulatory sequence that controls the binding of fVa to fXa and thus controls cofactor activity. For this purpose, we constructed several recombinant fV molecules to be assessed for their cofactor and clotting activities as well as for the direct binding to fXa. The results presented herein solidify the notion that only a short and discrete region from the B domain of fV is crucial and is required to keep the pro-cofactor in a quiescent state.

## EXPERIMENTAL PROCEDURES

### 

#### 

##### Materials

Diisopropyl fluorophosphate, *o*-phenylenediamine dihydrochloride, and Coomassie Blue R-250 were purchased from Sigma. fV-deficient plasma was from Research Protein Inc. (Essex Junction, VT). Secondary anti-mouse and anti-sheep IgG coupled to peroxidase was purchased from Southern Biotechnology Associates, Inc. (Birmingham, AL). l-α-Phosphatidylserine and l-α-phosphatidylcholine were obtained from Avanti Polar Lipids (Alabaster, AL). Chemiluminescent reagent ECL^+^ and heparin-Sepharose were obtained from Amersham Biosciences. Normal reference plasma and the chromogenic substrate *H*-d-hexahydrotyrosyl-alanyl-arginyl-*p*-nitroanilide diacetate (Spectrozyme-TH) were purchased from American Diagnostica Inc. (Greenwich, CT). RecombiPlasTin used in the clotting assays was purchased from Instrumentation Laboratory Co. (Lexington, MA). The reversible fluorescent α-thrombin inhibitor dansylarginine-*N*-(3-ethyl-1,5-pentanediyl)-amide (DAPA), human thrombin, human prothrombin, and active site-blocked human meizothrombin (obtained following digestion of prothrombin with the purified component from the venom of the snake *Echis carinatus*, FPR-meizothrombin) were purchased from Hematologic Technologies Inc. (Essex Junction, VT). Human fXa was purchased from Enzyme Research Laboratories (South Bend, IN). Human factor V cDNA was obtained from American Type Tissue Collection (ATCC 40515 pMT2-V, Manassas, VA). All restriction enzymes were obtained from New England Biolabs (Beverly, MA). All molecular biology and tissue culture reagents, specific primers, and medium were purchased from Invitrogen or as indicated. Recombinant prothrombin rMZ-II that has only one cleavage site for fXa (*i.e.* Arg^320^) and prothrombin rP2-II that has only one cleavage site for fXa (*i.e.* Arg^271^) were prepared and purified as described previously (24). Human fV monoclonal antibodies (αHFV_HC_17 and αHFV_LC_9) used for immunoblotting experiments and monoclonal antibody αHFV1 coupled to Sepharose used to purify plasma and recombinant factor V molecules were provided by K. G. Mann (Dept. of Biochemistry, University of Vermont, Burlington). Plasma fV (fV^plasma^) was purified as described previously (25, 26).

##### Construction of Recombinant fV Molecules

Recombinant fV molecules with mutations, fV^RQQ^, fV^Q3^, fV^ΔB9^, and fV^ΔB9/Q3^, were constructed using the QuikChange XL site-directed mutagenesis kit from Stratagene. We generated all cleavage activation mutants by changing arginine to glutamine. fV^Q3^ was constructed using the following mutagenic primers: 5′-GCA TTA GGA ATC CAG TCA TTC CGA AAC-3′ (sense) and 5′-GTT TCG GAA TGA CTG GAT TCC TAA TGC-3′ (antisense) (corresponding to the R709Q mutation); 5′-CCT TTA TCT CCG CAG ACC TTT CAC CCT C-3′ (sense) and 5′-G AGG GTG AAA GGT CTG CGG AGA TAA AGG-3′ (antisense) (corresponding to R1018Q mutation); and 5′-GCA TGG TAC CTC CAA AGC AAC AAT GG-3′ (sense) and 5′-CC ATT GTT GCT TTG GAG GTA CCA TGC-3′ (antisense) (corresponding to R1545Q mutation). fV^ΔB9^ was constructed using the mutagenic primers 5′-CTG AAG AAA AGC CAG TTT CTC ATT CAC ACA CAC CAT GCT CCT TTA TCT CCG-3′ (sense) and 5′-CGG AGA TAA AGG AGC ATG GTG TGT GTG AAT GAG AAA CTG GCT TTT CTT CAG-3′ (antisense) (corresponding to the ^1000^KTRKKKKEK^1008^ deletion). To construct fV^ΔB9/Q3^, the same primers were used, and fV^Q3^ was used as the template in the PCR. To construct fV^RQQ^, a similar methodology was employed with the mutagenic primers for R1018Q 5′-CCTTTATCTCCGCAGACCTTTCACCCTC-3′ (forward) and 5′-GAGGGTGAAAGGTCTGCGGAGATAAAGG-3′ (reverse) and the mutagenic primers for R1545Q 5′-GCATGGTACCTCCAAAGCAACAATGG-3′ (forward) and 5′-CCATTGTTGCTTTGGAGGTACCATGC-3′ (reverse). All mutations were confirmed by DNA sequencing (DNA Analysis Facility, Dept. of Molecular Cardiology at The Lerner Research Institute, Cleveland Clinic, Cleveland, OH).

##### Transient Transfection, Purification, and Assay of Recombinant fV Molecules

Purified wild type fV (fV^WT^), fV^ΔB9^, fV^ΔB9/Q3^, fV^RQQ^, and fV^Q3^ plasmids were transfected into COS-7L cells with FuGENE 6 (Roche Diagnostics), according to the manufacturer's instructions as described extensively by our laboratory (17, 18). A 4-ml column of monoclonal antibody αHFV1 coupled to Sepharose was used to purify recombinant proteins as extensively described previously (27). To avoid repeated freeze-thaw cycles, small aliquots of the purified proteins were stored at −80 °C. Clotting assays using fV-deficient plasma and Western blotting with monoclonal and polyclonal antibodies were used to determine the activity and integrity of the recombinant fV molecules as described previously (25, 28). Thrombin was used for the activation of fV and was inhibited by the addition of PMSF. Any residual thrombin used in activation that could potentially contribute to our experimental data were eliminated in the reaction mixtures by the addition of DAPA. All experimental procedures were thoroughly and extensively described previously (17, 18, 27, 29).

##### Determination of the Concentration of the Recombinant fV Molecules

The concentration of fV was determined with an ELISA as extensively described (17, 18, 27, 28). The absorbance at 490 nm was monitored using an Amersham Biosciences THERMOMAX microplate reader. Because of slight differences in time of incubation with the substrate, in every experiment a plasma fV standard (serial dilutions of purified plasma fV) was run, and all values obtained with the recombinant molecules were compared with the plasma fV standard values within the same 96-well plate. No comparison in concentration was made between recombinant molecules from one plate to another. The determination of the concentration of the recombinant molecules was performed by averaging the value found for each sample run in triplicate.

##### Gel Electrophoresis and Western Blotting

SDS-PAGE analyses were carried out using 4–12% gradient gels or 9.5% gels following reduction with 2% β-mercaptoethanol, according to the method of Laemmli, and the protein was visualized by being stained with Coomassie Brilliant Blue R-250, followed by destaining in a solution of methanol, acetic acid, and water (30). In several experiments, the protein was transferred to polyvinylidene difluoride (PVDF) membranes following a modification of the method described by Towbin *et al.* (31). The membrane transferred proteins were incubated with monoclonal and polyclonal antibodies specific to fV heavy/light chains, and the protein was visualized by immunoreactive chemiluminescence using ECL Plus reagents (GE Healthcare).

##### Kinetic Titrations of Prothrombinase

A discontinuous assay was used to assess the ability of the recombinant fV molecules to assemble in the prothrombinase complex and bind to the enzyme (18). Briefly, fV variants were assayed after (fVa) and before (fV) the activation with thrombin. To determine the dissociation constant (*K_D_*_(app)_) between the fV/fVa molecules and fXa, we prepared separate reaction mixtures that contained PCPS vesicles (where PCPS indicates small unilamellar phospholipids vesicles composed of 75% l-α-phosphatidylcholine and 25% l-α-phosphatidylserine (w/w)) (20 μm), DAPA (3 μm), a limiting fXa concentration (15 pm) and varying concentrations of the recombinant fVa species (30, 60, 125, 250, and 500 pm and 1, 2.5, 5, and 10 nm). The reaction was initiated with a constant and saturating amount of prothrombin (1.4 μm). At selected time intervals into each reaction (20, 40, and 60 s), aliquots were added separately to 80 μl of quench buffer in a 96-well microtiter plate, and the primary thrombin plots (the rate of thrombin generation as a function of time) were measured using Spectrozyme-TH (0.4 mm). The *K_D_*_(app)_ of each recombinant fVa species for fXa was then calculated by plotting the data as thrombin activity (nm IIa/min) as a function of the fV/fVa concentration to the equation representing a one binding site model using the software Prism® (GraphPad, San Diego). All experiments were performed at least in duplicate, and the goodness of fit (*R*^2^) for the model (equation) tested is provided in each table. In addition, and to avoid any artifacts or inter-experimental variation, all recombinant mutant fV/fVa molecules were assayed at the same time and with the same reagents as the wild type or plasma cofactor molecule, on the same 96-well microtiter plate. The *K_D_*_(app)_ obtained directly from the graphs was used to calculate the amount of fV/fVa necessary to saturate fXa using the quadratic equation extensively described in detail elsewhere (32–34).

To determine the kinetic constants (*K_m_* and *k*_cat_) of prothrombinase, assays were performed using limited amounts of fXa (10 pm) in the presence of a fixed (saturating) amount of various recombinant fV/fVa molecules (20–80 nm) while varying the prothrombin concentration. The reaction was initiated by adding increasing concentrations of prothrombin (25, 50, 100, 250, 500, and 750 nm and 1, 2, and 4 μm) to the mixture already containing a constant amount of prothrombinase determined as described above to be ∼10 pm using the known *K_D_*_(app)_ for each variant and the quadratic equation described previously (32, 33). At selected time intervals into each reaction, aliquots were added separately to 80 μl of quench buffer in a 96-well microtiter plate, and the primary thrombin plots (initial rate of thrombin generation) were obtained with the chromogenic substrate Spectrozyme-TH. The data were plotted to the Michaelis-Menten equation as nm IIa/min as a function of prothrombin and analyzed with the software Prism®. All experiments were performed at least in duplicate, and the goodness of fit (*R*^2^) for the model tested (Michaelis-Menten equation) is provided in each table. To avoid any artifacts or inter-experimental variation, all recombinant mutant fV/fVa molecules were assayed at the same time and with the same reagents as the wild type or plasma cofactor molecule, on the same 96-well microtiter plate. All kinetic constants reported in the tables were derived directly from the graphs.

##### Stabilization of the Transition State

Prothrombinase is an enzyme composed of fVa (regulatory subunit) and fXa (catalytic subunit) assembled on a membrane surface. Any perturbation in the interaction between the two molecules caused by a mutation may influence or modify the stability of the catalytic site of the enzyme and can be measured by the change in the transition-state stabilization of free energy for prothrombin activation as described (35–39). Thus, the consequence of the mutations in fVa affecting fXa catalytic efficiency can be measured relative to the change in transition-state stabilization free energy (ΔΔ*G*^‡^) of the enzyme. To assess whether the deletion in the B region affects the stabilization of the transition state, separate free energies associated with the catalytic efficiency of prothrombinase must be measured as follows: Δ*G*_WT_, the functional free energy in prothrombinase assembled with fVa^WT^; Δ*G*_ΔB9/Q3_, the functional free energy in prothrombinase assembled with fV^ΔB9/Q3^; and Δ*G*_RQQ_, the functional free energy in prothrombinase assembled with fVa^RQQ^. The perturbation to the function of the enzyme (prothrombinase) caused by a mutation in its regulatory subunit (fVa) affecting the transition state can be defined as shown in Equations 1 and 2,

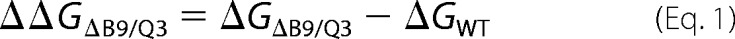


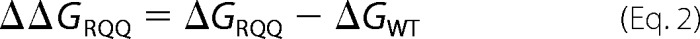
 The specificity constant (also known as catalytic efficiency or second-order rate constant), which is defined by the ratio *k*_cat_/*K_m_*, does not measure the rate of an enzyme *per se*, but rather it measures the efficiency of an enzyme toward different substrates or the efficiency of different enzymes or mutant enzyme molecules *versus* a common substrate (36–38). Thus, it is generally used to evaluate the effectiveness of different enzymes to one another when assessed against the same substrate. Because we are measuring prothrombinase activity assembled in the presence of various fV/fVa molecules against the same substrate (prothrombin), the changes in transition-state stabilization free energy (ΔΔ*G*^‡^) during catalysis caused by the mutations in fV/fVa can be calculated from Equations 3 and 4,

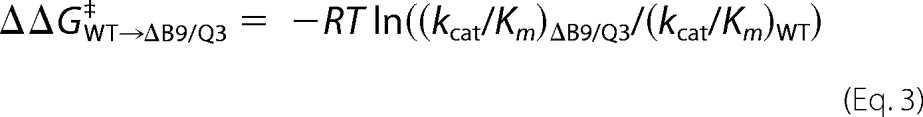


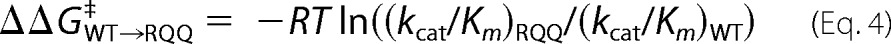
 where *R* is the universal gas constant (2 cal· K^−1^·mol^−1^); *T* is the absolute temperature in Kelvin (298 K in all our experiments); *k*_cat_ is the turnover number, and *K_m_* is the Michaelis-Menten constant for prothrombinase assembled with either wild type fVa or mutant fV/Va molecules.

##### Studies of the Pathway for Prothrombin Activation by Gel Electrophoresis

Reaction mixtures were incubated with prothrombin containing 20 μm PCPS, 8 μm DAPA, and various concentrations of fVa^WT^, fVa^ΔB9^, and fVa^RQQ^ (incubated with thrombin, 2 nm) and fV^Q3^ and fV^ΔB9/Q3^ in TBS with Ca^2+^. Before the addition of fXa (0.5 nm) and the start of the reaction, a zero point was taken. Aliquots of the reaction mixture were removed at selected time points and treated as described previously in detail (18, 40, 41). Prothrombin consumption was visualized by gel electrophoresis and quantified by using densitometry as described previously (40, 41). For the studies of prothrombin cleavage and activation by factor Xa alone, a similar protocol was used as described (41). Briefly, reaction mixtures containing 1.4 μm prothrombin, 20 μm PCPS, and 8 μm DAPA were incubated for 5 min, and the reaction was initiated with the addition of factor Xa (0.5–1 nm) at room temperature over a 1-h time course. Aliquots (50 μl) from the reaction were removed at selected time intervals (as indicated in the legend to the figures), treated as described previously (40), and analyzed using 9.5% SDS-PAGE. Prothrombin consumption rates were calculated as described previously in detail (40, 41).

##### Studies to Control Recombinant fV Back Activation during Prothrombin Activation Assays

Wild type fVa and mutant fV/fVa molecules were assessed for activation throughout a prothrombin activation assay as described. Before the incubation with fXa (0.5 nm) into the reaction mixture, a zero point was taken. A sample was also taken following a 1-h incubation with fXa and all the components of prothrombinase. Aliquots of the reaction mixture containing recombinant fV/fVa molecules (1 μg) were removed and immediately diluted into 2-fold the volume of 0.2 m acetic acid and concentrated by centrifugation as described (40). The pellets were dissolved in a 0.1 m Tris base, pH 6.8, and 1% SDS. After reconstitution, the samples were subjected to 4–12% gradient gels and probed for activation fragments using Western blotting techniques as described previously (41, 42).

## RESULTS

### 

#### 

##### Transient Expression and Analysis of Recombinant fV Molecules

**FIGURE 3.**
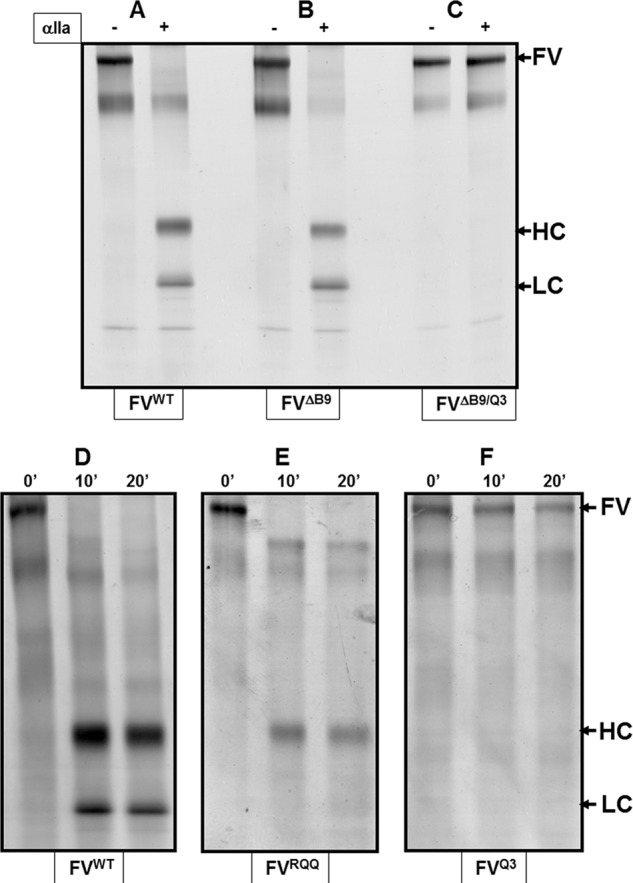
**Electrophoretic analyses of the purified recombinant molecules.** Purified recombinant fV^WT^ and purified recombinant fV molecules fV^ΔB9^, fV^ΔB9/Q3^, fV^RQQ^, and fV^Q3^ were incubated with thrombin as described under “Experimental Procedures” and analyzed by SDS-PAGE followed by staining with silver. *Panels A–C* show the molecules before (−) and after activation by thrombin (+). *Panels D–F* show the molecules before (0 min) and after incubation with thrombin for 10 and 20 min. The identity of each fV molecule is shown *below* each panel. *HC*, heavy chain; *LC*, light chain.

To investigate the importance of amino acid region 1000–1008 of the B domain of fV, we constructed four mutant molecules. We prepared recombinant fV^RQQ^, fV^ΔB9^, fV^Q3^, and fV^ΔB9/Q3^. All recombinant molecules were expressed as full-length derivatives in mammalian cells and were purified to homogeneity by immunoaffinity chromatography as described previously in great detail by our laboratory (27). A typical routine quality control method performed in our laboratory before all experiments is to assess recombinant fV^WT^ and mutant molecules for their integrity following activation by thrombin. Fig. 3 illustrates the composition of the recombinant molecules before and after incubation with thrombin following staining with silver. To rule out any contribution of minute impurities to cofactor activity, we compared the clotting activities of fVa^WT^ and fVa purified from pooled fresh frozen normal plasma (fVa^plasma^). All data obtained from purified fVa^plasma^ and recombinant fVa^WT^ were comparable. fVa^WT^ activated with thrombin had similar cofactor activity when compared directly with fVa^plasma^ (Table 1). It is also important to note that the data obtained with purified recombinant fVa^WT^ is comparable with recombinant fVa molecules used in conditioned media (17, 18). Thus, any small impurities that may be present in our fV preparations do not have any impact on cofactor activity. Additionally, purified recombinant fV molecules with all activation sites changed to glutamine are unable to be cleaved and form active cofactors (Fig. 3, *panels C* and *F*). Finally, the activation quotient of fV^Q3^ that measures the ratio of activity after and before deliberate activation by thrombin was close to 18, demonstrating very little unintentional activation of fV^Q3^ during the purification procedures (25).

**TABLE 1:** **Functional properties of recombinant fV molecules**

Factor Va species	Clotting activity*^a^*	Decrease*^b^*	*K_D_*_(app)_*^c^*	*R*^2^/points studied for *K_D_*_(app)_*^d^*	*K_m_**^e^*	*k*_cat_*^e,f^*	*k*_cat_/*K_m_*	Decrease*^g^*
	*units/mg*	*-fold*	*nm*		μ*m*	*min*^−*1*^	*m*^−*1*^·*s*^−*1*^ × *10^6^*	*-fold*
fVa^WT^	3590 ± 300		0.28 ± 0.03	0.92/80	0.14 ± 0.01	1993 ± 36	237	
fVa^plasma^	3200 ± 274	1.1	0.51 ± 0.06	0.99/10	0.17 ± 0.02	2030 ± 45	199	1.2
fV^Q3^	206 ± 37	17.4	5.5 ± 7.15	0.37/30				
fV^ΔB9/Q3^	3110 ± 65	1.1	0.73 ± 0.08	0.93/80	8.0 ± 3.3	452 ± 140	0.94	252
fVa^RQQ^	2370 ± 465	1.5	0.92 ± 0.27	0.90/20	0.18 ± 0.04	655 ± 33	61	4

*^a^* Two-stage clotting assays of recombinant fV molecules were performed as described under “Experimental Procedures.”

*^b^* The -fold decrease is the ratio of the clotting activity of fVa^WT^ compared with the clotting activity of all other fV molecules. The ratio of clotting activity between fVa^WT^ and fV^Q3^ demonstrates that there is very little unintentional activation of fV^Q3^ (*AQ* = 17.4) (25).

*^c^* The apparent dissociation constants of recombinant fV/fVa and plasma fVa molecules for plasma-derived fXa (*K_D_*_(app)_) were determined as described under “Experimental Procedures” at limiting fXa concentrations (15 pm) according to the binding model assuming one binding site using the software Prism®. Apparent dissociation constants were derived directly from the fitted data. The data shown represent average numbers from eight separate experiments performed with eight different preparations of fVa^WT^, eight different preparations of fV^ΔB9/Q3^, three different preparations of fV^Q3^, and one preparation of fVa^plasma^.

*^d^ R*^2^ represents the goodness of fit to the equation describing the binding model assuming one binding site using the software Prism®. Points studied represent eight separate graphs (10 measurements/graph) for experiments using fVa^WT^ and fV^ΔB9/Q3^ employing eight different preparations of recombinant fV molecules. The *K_D_*_(app)_ of fV^Q3^ for fXa was obtained using three separate graphs with three different preparations of fV^Q3^, whereas the *K_D_*_(app)_ of fVa^plasma^ for fXa was obtained from one graph using one preparation of fV^plasma^.

*^e^* The *K_m_* and *k*_cat_ values of prothrombinase assembled with saturating concentrations of plasma fVa and recombinant fV/fVa molecules were determined as described under “Experimental Procedures” according to the Michaelis-Menten equation using the software Prism®. Kinetic constants were derived directly from the fitted data shown in Fig. 6.

*^f^ k*_cat_ = *V*_max_/[enzyme] (in the presence of fVa); the enzyme concentrations of prothrombinase (fXa-fV/fVa complex on the membrane surface in the presence of Ca^2+^) were calculated based on the equations previously described in detail in the literature. Under the conditions employed herein, prothrombinase concentration was assumed to be ∼10 pm (the fXa used was >98% saturated with fVa).

*^g^* The -fold decrease is the ratio of the specificity constant (*k*_cat_/*K_m_*) of prothrombinase assembled with fVa^WT^ compared with the specificity constant of prothrombinase assembled with fV^ΔB9/Q3^.

As shown in Fig. 3, recombinant fV^ΔB9/Q3^ and fV^Q3^ molecules having all activation sites changed to glutamine (*panels C* and *F,* respectively) were resistant to thrombin cleavage and activation, whereas fV^WT^ and fV^ΔB9^ following incubation with thrombin exhibited the normal fragments representing the heavy and light chains of the active cofactor (*panels A, B,* and *D*). In addition, fV^RQQ^ produced only the expected heavy chain of the cofactor and an *M*_r_ 220,000 intermediate following extended incubation with thrombin (Fig. 3, *panel E*). A two-stage clotting assay was first used to assess cofactor activity of the recombinant molecules. Using similar experimental conditions, fV^Q3^ was found to have clotting activity 17.4-fold lower than fVa^WT^. In addition, although fV^Q3^ is severely impaired in its clotting activity, unexpectedly fV^ΔB9/Q3^ had clotting activity comparable with fVa^WT^ and fVa^RQQ^ (Table 1). These data demonstrate that amino acid region 1000–1008 is important for the regulation of optimal clotting activity of fVa. These data also show that cleavage at Arg^709^ alone is sufficient to recover most fVa clotting activity.

##### Kinetic Analyses of Recombinant fV Molecules

We have observed thus far that deletion of amino acid region 1000–1008 from the B region of fV results in a molecule that does not require prior activation by thrombin to acquire optimum clotting activity. In contrast, a recombinant molecule without the deletion but with all activation sites changed to glutamine (fV^Q3^) was practically devoid of cofactor activity. It is thus possible that the nine amino acids deleted in this molecule (fV^ΔB9/Q3^) are required to keep fV in a quiescent state and thus impair the high affinity fVa-fXa interaction on the membrane surface required for prothrombinase assembly and function. As a consequence, the ability of recombinant fV molecules to bind fXa was studied next using purified reagents and a chromogenic substrate assessing thrombin formation. The assay was performed under conditions of varying concentrations of recombinant fV/fVa molecules while using limiting concentrations of fXa. This assay has been widely used in the coagulation field to determine the *K_D_*_(app)_ value for the fVa-fXa or factor VIIIa-factor IXa interactions when the concentration of the cofactors is sparse (recombinant molecules or plasma-derived cofactors from various fV variants) (20, 21, 43–46).

**FIGURE 4.**
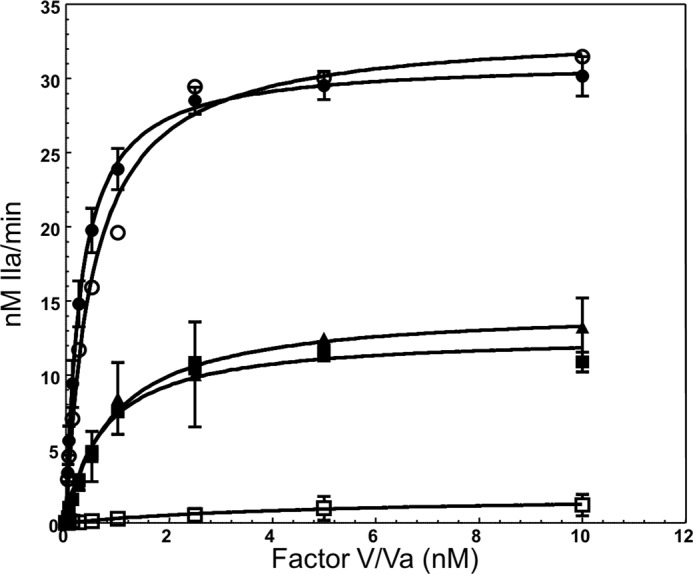
**Determination of the dissociation constant of recombinant fV^ΔB9/Q3^ and fV^Q3^ for plasma fXa.** Thrombin generation experiments were carried out as described under “Experimental Procedures.” Prothrombinase assembled with varying concentrations of recombinant purified fV^Q3^ is depicted by *open squares*, prothrombinase assembled with purified recombinant fV^ΔB9/Q3^ by *filled squares*, and prothrombinase assembled with purified recombinant fVa^RQQ^ by *filled triangles.* Prothrombinase assembled with varying concentrations of recombinant purified fVa^WT^ is depicted by *filled circles*, and prothrombinase assembled with purified recombinant fVa^plasma^ is depicted by *open circles*. The *solid lines* represent a nonlinear regression fit of the data using Prism® GraphPad software and the equation describing the one binding site model. Titrations shown herein were performed with multiple preparations of recombinant proteins as detailed in Table 1. The kinetic constants derived directly from the plotted data are also reported in Table 1. The assay was performed with fV/fVa species varying from 0 to 15 nm. However, for the easy plotting of the data, only points from 0 to 10 nm are shown.

The results from the binding studies are shown in Fig. 4, and the data extracted directly from the plots are reported in Table 1. Using this methodology, we have repetitively shown that the *K_D_*_(app)_ value of the bimolecular interaction between fVa and fXa is similar to the *K_D_*_(app)_ value established by several other binding methods (6, 14, 18, 22, 47–49). The data demonstrate that fV^Q3^ was unable to interact with fXa (Fig. 4, *open squares*, and Table 1). In contrast, the affinity of recombinant mutant fV^ΔB9/Q3^ for plasma-derived fXa was similar to the affinity of fVa^WT^ or fVa^plasma^ for the plasma-derived enzyme (Table 1 and Fig. 4, *filled squares* and *filled* and *open circles,* respectively). These results contradict recent findings (49) and demonstrate that only a very limited stretch of nine amino acids within the B domain of fV is required to sheath the interactive site(s) within fV that are essential for its binding to plasma-derived fXa and as a consequence keep the pro-cofactor in a quiescent state. Direct comparison between the data obtained with prothrombinase assembled with fVa^WT^, fVa^RQQ^, and fV^Q3^ demonstrates that under similar experimental conditions the initial cleavage at Arg^709^ and the generation of the heavy chain alone resulted in a molecule (fVa^RQQ^) that has a similar affinity for plasma fXa as fVa^WT^ or fVa^plasma^ (Fig. 4, *filled triangles*, and Table 1).

We next performed a titration of fXa with fV^ΔB9/Q3^. An increase in the concentration of fV^ΔB9/Q3^ within prothrombinase resulted in an increase in both the *K_m_* and the *k*_cat_ values of the enzymatic reaction, although the effective enzyme concentration remained approximately constant (Table 2). In addition, the specificity constant varied very little over a wide range of concentrations of fV^ΔB9/Q3^. This fact is obvious when comparing the results obtained with prothrombinase made with 80 nm fV^ΔB9/Q3^ with the data obtained by using prothrombinase assembled with either 15 or 30 nm fV^ΔB9/Q3^ (Table 2). The increase in the *k*_cat_ (*V*_max_/*E_T_*) of the enzymatic reaction obtained with prothrombinase assembled with fV^ΔB9/Q3^ that is concomitant with an increase in *K_m_* can be easily explained (assuming that *K_m_* ∼ *K_s_*) by the fact that at any given point, under the experimental conditions employed, the effective concentration of free enzyme in solution is maximized when the enzyme (prothrombinase) is assembled with fV^ΔB9/Q3^ compared with the concentration of free enzyme in solution when prothrombinase is assembled with fVa^WT^ (36). As a consequence, at any given time during the reaction as much as possible of prothrombinase assembled with fV^ΔB9/Q3^ exists in the unbound form compared with prothrombinase made with fVa^WT^. Altogether, the data demonstrate that the stretch of amino acids 1000–1008 from the B region of the pro-cofactor keeps the molecule in a quiescent state.

**TABLE 2:** **Kinetic parameters of prothrombinase with increasing concentrations of purified mutant recombinant fV molecules**

Recombinant fV species	fXa saturation*^a^*	*R*^2^/titrations*^b^*	*K_m_*	*k*_cat_	Effective prothrombinase concentration*^c^*	*k*_cat_/*K_m_*
*nm*	%		μ*m*	*min*^−*1*^	*pm*	*m*^−*1*^·*s*^−*1*^ × *10^6^*
fV^ΔB9/Q3^ (15)	95.3	0.93–0.95/3	0.5 ± 0.3	122 ± 16	9.5	4.1
fV^ΔB9/Q3^ (30)	97.6	0.9–0.95/3	1.63 ± 0.4	207 ± 55	9.8	2.1
fV^ΔB9/Q3^ (60)*^d^*	98.8	0.94–0.99/5	9 ± 3.7	487 ± 165	9.9	0.9
fV^ΔB9/Q3^ (80)	99.0	0.93–0.97/2	12 ± 8.3	866 ± 480	9.9	1.2
fVa^RQQ^ (20)	95.3	0.90/2	0.18 ± 0.04*^e^*	655 ± 33*^e^*	9.5	61*^e^*
fVa^RQQ^ (60)	98.5	0.95/1	0.15 ± 0.04	821 ± 51	9.8	91

*^a^* The percent saturation was calculated using a quadratic equation as described under “Experimental Procedures,” using a *K_d_* of 0.73 and 0.92 nm between fV^ΔB9/Q3^ or fVa^RQQ^ and fXa, respectively, and 10 pm fXa for all assays.

*^b^ R*^2^ represents the goodness of fit (minimum and maximum) for all titrations to the equation describing Michaelis-Menten kinetics using the software Prism®. Titrations studied represent three separate graphs (10 measurements/graph) for experiments using 15 and 30 nm fV^ΔB9/Q3^ and five different titrations (10 measurements/graph) using 60 nm fV^ΔB9/Q3^.

*^c^* The effective prothrombinase concentrations were obtained by multiplying the fXa concentration used in each titration by the saturation factor.

*^d^* All titrations were performed with five different recombinant fV preparations. These preparations were different from the preparations used to obtain the data shown in Table 1.

*^e^* Value is from Table 1.

It is important to note that the *K_m_* and *k*_cat_ values of prothrombinase assembled with fV^ΔB9/Q3^ vary (increase) with the concentration of mutant pro-cofactor used in the assay to assembly prothrombinase (Table 2). At 15 nm fV^ΔB9/Q3^, the *K_m_* value of prothrombinase for prothrombin is 0.5 μm (Table 2), whereas the *K_m_* value of prothrombinase assembled with fVa^WT^ and fVa^RQQ^ under similar conditions is 0.15 and 0.18 μm, respectively (Table 1). In the presence of 30 nm fV^ΔB9/Q3^, the *K_m_* value of prothrombinase for prothrombin is 1.2 μm, and at 60 nm the *K_m_* value of prothrombinase for prothrombin is 8 μm. Fig. 4 illustrates an assay where the concentration of fVa^WT^/fVa^RQQ^/fV^ΔB9/Q3^ varies between 0 and 15 nm. Under these conditions, the *K_m_* value of prothrombinase assembled with fV^ΔB9/Q3^ is 0.5 μm, whereas the *K_m_* value of prothrombinase assembled with fVa^WT^ or fVa^RQQ^ is 0.15 and 0.18 μm, respectively. Thus, considering the Michaelis-Menten Equation 5,

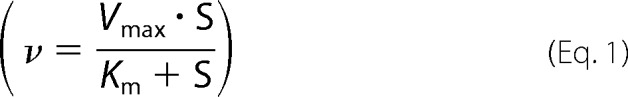
 in the presence of prothrombinase assembled with fV^ΔB9/Q3^ (with prothrombin used at 1.4 μm, which is ∼3 *K_m_*), the rate of the reaction is 75% (±10%) of the maximum rate. Any further increase in S gives only a marginal increase in the rate of the reaction. In the case of prothrombinase assembled with fVa^RQQ^ (with prothrombin used at 1.4 μm, which is ∼7 *K_m_*), the rate of the reaction is 87% of the maximum rate, although a similar calculation using fVa^WT^ reveals that the rate of the reaction is 90% of the maximum rate (*V*_max_). Thus, any small variation in the interaction of prothrombinase with prothrombin (*K_m_* ∼0.15–0.5 μm) is rendered meaningless because all species are incubated with an excess amount of prothrombin (1.4 μm). As a result, the binding assay comparing all prothrombinase enzymes tested in Fig. 4 is kinetically sound and provides compelling data on the fV/fVa-fXa association because the enzymes assembled with various forms of recombinant fV/fVa molecules work at similar rates.

**FIGURE 5.**
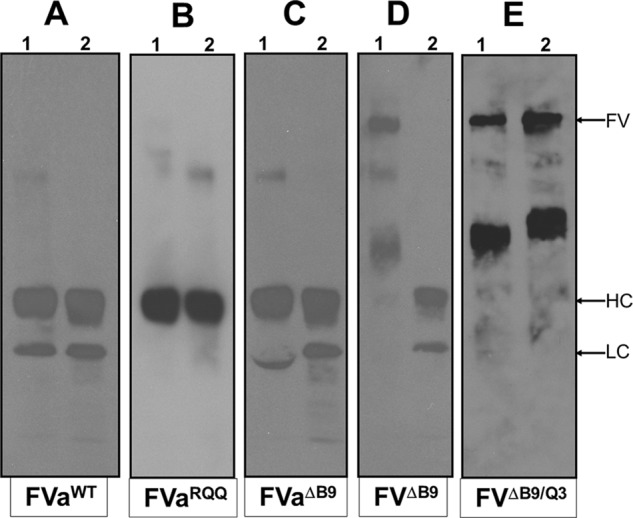
**Analyses of back activation of wild type fV and recombinant fV molecules within a prothrombinase assay.** fVa^WT^, fVa^ΔB9^, fV^ΔB9^, fVa^RQQ^, and fV^ΔB9/Q3^ were incubated in a prothrombin activation assay mixture before (*lane 1*) and 1 h after (*lane 2*) the addition of fXa as described under “Experimental Procedures.” The reaction was stopped as described under “Experimental Procedures.” After SDS-PAGE and transfer to a PVDF membrane, fV fragments were detected using monoclonal antibodies αHFVa_HC_17 and αHFVa_LC_9 recognizing the heavy chain (*HC*) and light chain (*LC*), respectively. At *right*, the positions of fV and the heavy and light chains of fVa are shown.

Our assumption thus far is that fV^ΔB9/Q3^ within each mixture remains intact for the duration of the experiments. However, during the experiments reported above, we are assessing the fV^ΔB9/Q3^ interaction with fXa in the presence of membrane-bound fXa in an assay that measures thrombin formation. Although we are using an excess of a specific thrombin inhibitor, and although all fV activation cleavage sites are changed to glutamine, it is important to assess the integrity of the fV^ΔB9/Q3^ molecule within the mixture containing all reagents, prior to and at the end of the experiment to verify for any incidental cleavage and/or activation of the molecule during the experiments. Fig. 5 shows the appropriate control experiments. The data demonstrate that although fV^ΔB9^ is indeed activated during the course of the assay (Fig. 5, *panel D, lane 2*), fV^ΔB9/Q3^ remains intact during the entire duration of the experiment (Fig. 5, *panel E, lane 2*). A direct comparison of the data shown in Fig. 4 (*open* and *closed squares*) with the data illustrated in Fig. 5 (*panel E*) demonstrates that the high affinity interaction between fV^ΔB9/Q3^ and fXa can only be attributed to the deletion of amino acid region 1000–1008, because fV^Q3^ under similar experimental conditions has no measurable affinity for fXa. It is noteworthy that although a 20-fold lower *K_D_*_(app)_ value of fV^Q3^ for plasma-derived fXa is reported in Table 1, the goodness of fit to the equation describing the one binding site model is very low (*R*^2^ of 0.37, Table 1) suggesting that under the experimental conditions used fV^Q3^ most likely does not interact with fXa. Overall, the data clearly indicate that the high affinity specific interaction between fV^ΔB9/Q3^ and fXa is a direct consequence of the deletion of the nine amino acids from the B region of the pro-cofactor.

**FIGURE 6.**
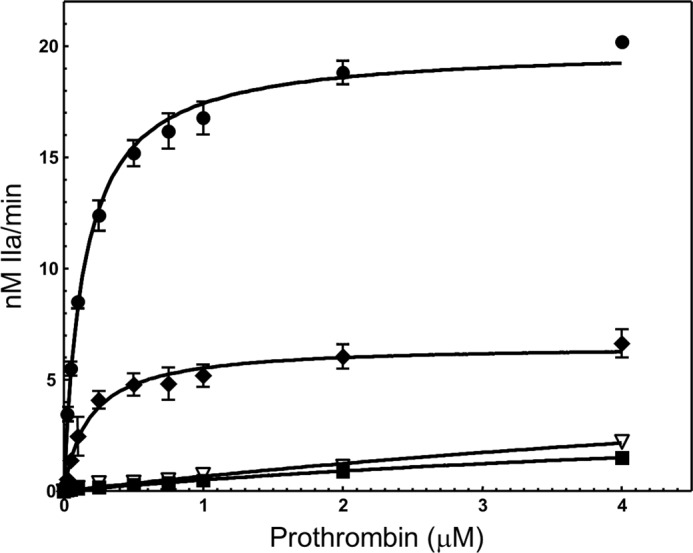
**Determination of kinetic parameters of prothrombinase assembled with various fV/Va species.** Initial rates of thrombin generation were determined using the *K_D_*_(app)_ for fXa found in Fig. 4 to determine the concentration of fVa necessary to obtain over 98% fXa saturation as described under “Experimental Procedures” in the presence of PCPS vesicles and fXa (32, 33). Data for prothrombinase assembled with fVa^WT^ are shown by *filled circles* (20 nm, 98.6% fXa saturation). Data for prothrombinase assembled with 60 nm fV^ΔB9/Q3^ are depicted by *filled squares* (98% fXa saturation), and data for prothrombinase assembled with 80 nm fV^ΔB9/Q3^ are depicted by *open inverse triangles* (99% fXa saturation). Data for prothrombinase assembled with fVa^RQQ^ are shown by *filled diamonds*. The titration shown with 60 nm fV^ΔB9/Q3^ is the average of results from experiments performed with five different preparations of purified recombinant protein, although the titration with 80 nm fV^ΔB9/Q3^ is the average from two different preparations of recombinant protein. The kinetic constants were derived directly from the plotted data and are reported in Tables 1 and 2.

Subsequently, we evaluated the recombinant fV/fVa molecules for their ability to assemble in prothrombinase and activate prothrombin under similar experimental conditions (saturating fV/fVa concentrations with respect to fXa (>98%)). Table 1 shows all kinetic constants derived directly from the fitted data for each set of titrations (Fig. 6). Prothrombinase assembled with saturating concentrations of recombinant fV^ΔB9/Q3^ (60 nm, >98% saturation) had a *K_m_* value that was 57-fold higher than the *K_m_* value of prothrombinase assembled with saturating concentrations of either fVa^plasma^ or fVa^WT^ (20 nm, >98% saturation) for prothrombin (Table 1). In contrast, under the conditions employed, the *k*_cat_ (turnover number) of prothrombinase assembled with fV^ΔB9/Q3^ was decreased by a modest 4-fold (Table 1) resulting in an overall 252-fold decrease of the specificity constant of the reaction when compared with the specificity constant obtained with prothrombinase assembled with fVa^WT^ (Table 1). This particular phenomenon occurring when *K_m_* > [S], so that the *E*S complex is at a higher energy level than [*E*] + [S], has been already described (36, 38) and demonstrates that, under these precise conditions, the *V*_max_ of an enzymatic reaction increases as the interaction of the substrate with the enzyme becomes weaker in the transition state (Table 2) (36, 38, 50). This fact is obvious when using the values provided in Table 1 and Equation 3. A difference in transition state free energy of 3.3 kcal/mol can be observed between prothrombinase assembled with fV^ΔB9/Q3^ and prothrombinase assembled with fVa^WT^ consistent with a strong interaction between the latter enzyme and prothrombin in the transition state. Interestingly, although the *k*_cat_ of prothrombinase assembled with fVa^RQQ^ is modestly decreased by 3-fold, the *K_m_* value of the reaction is similar to the *K_m_* value obtained with fVa^WT^, resulting in an overall 3-fold decrease in the specificity constant of the enzyme (Table 1). The ratio of the *k*_cat_/*K_m_* between prothrombinase assembled with fVa^RQQ^ and prothrombinase made with fVa^WT^ (Table 1) from Equation 4 corresponds to a modest difference in transition state free energy of 0.81 kcal/mol, suggesting that there is no significant difference in the binding of the transition state intermediate between prothrombinase assembled with fVa^WT^ and prothrombinase made with fVa^RQQ^. Thus, like prothrombinase made with fVa^WT^, prothrombinase assembled with fVa^RQQ^ established a strong interaction with the substrate in the transition state resulting in similar (low) *K_m_* values. A simple comparison of the data obtained with prothrombinase assembled with fVa^WT^, fVa^RQQ^ (Table 2), or fV^Q3^ demonstrates that under similar experimental conditions the cleavage at Arg^709^ alone is required and sufficient to promote optimum interaction of prothrombin with prothrombinase (lower *K_m_*).

##### Visualization of the Activation Pathway

**FIGURE 7.**
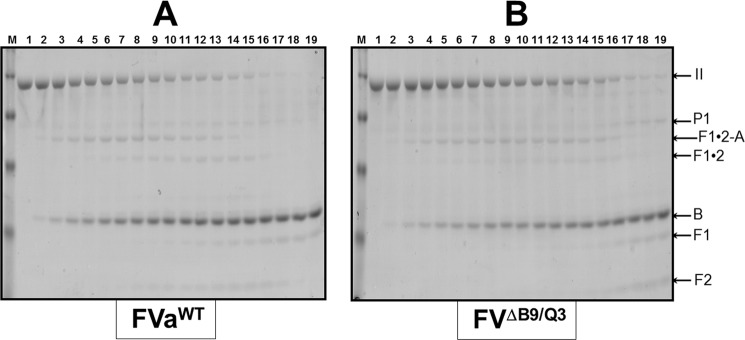
**SDS-PAGE analyses of prothrombin-activated fragments by prothrombinase assembled with various recombinant molecules.** *Panel A,* plasma-derived prothrombin (1.4 μm) was activated by prothrombinase assembled with fVa^WT^ (final concentration of 20 nm, 96.6% fXa saturation); *panel B,* prothrombinase assembled with fV^ΔB9/Q3^ (final concentration of 60 nm, 99% fXa saturation). Aliquots were withdrawn at various time intervals and treated as described (18, 40). *M* represents the lane with molecular weight markers (from *top* to *bottom*): 98,000, 64,000, 50,000, and 36,000, respectively. *Lanes 1–19* show samples from the reaction mixture before (0 min) the addition of fXa and 20, 40, 60, 80, 100, 120, 140, 160, 180, 200, 220, and 240 s and 5, 6, 10, 20, 30, and 60 min, respectively, after the addition of fXa. Positions of prothrombin-derived fragments are shown to the *right* of *panel B*.

Our findings thus far specify that fV^ΔB9/Q3^ is fully able to bind the enzyme fXa with high affinity and induce clotting in fV-deficient plasma. Prothrombinase assembled with fV^ΔB9/Q3^ is also capable of activating prothrombin with a *k*_cat_ (*V*_max_/*E_T_*) that is only 4-fold slower than the *k*_cat_ for the reaction produced by prothrombinase assembled with fVa^WT^. However, in an assay using purified reagents, there is a substantial increase in the Michaelis-Menten constant (*K_m_*) of prothrombinase assembled with fV^ΔB9/Q3^ compared with prothrombinase assembled with fVa^WT^. To better understand these results and to establish if there is a defect in the pathway for prothrombin activation by prothrombinase assembled with fV^ΔB9/Q3^, we studied prothrombin activation by gel electrophoresis (Fig. 7).

**FIGURE 8.**
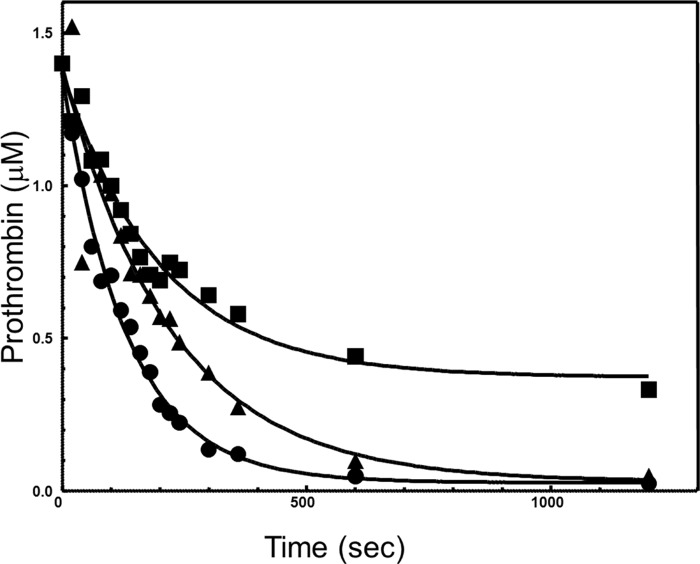
**Analysis of prothrombin consumption by prothrombinase assembled with recombinant fV/fVa molecules.** The two gels shown in Fig. 7 were scanned, and prothrombin consumption was recorded as described under “Experimental Procedures.” Following scanning densitometry, the data representing prothrombin consumption as a function of time (seconds) were plotted using nonlinear regression analysis according to the equation representing a first-order exponential decay using the software Prism®. The apparent first-order rate constant, *k* (s^−1^), was obtained directly from the fitted data. Prothrombinase was assembled with recombinant fVa^WT^ (*filled circles*), fV^ΔB9/Q3^ (*filled squares*), and factor Va^RQQ^ (gel not shown in Fig. 7, *filled triangles*).

The results demonstrate a modest delay in plasma prothrombin activation by prothrombinase assembled with fV^ΔB9/Q3^ as compared with the activation of prothrombin by prothrombinase assembled with fVa^WT^ (Fig. 7). These data are in complete accord with the kinetic data. Scanning densitometry of the gels shown in Fig. 7 demonstrated a 1.5-fold delay in prothrombin consumption by prothrombinase assembled with fV^ΔB9/Q3^ as compared with the consumption of prothrombin by prothrombinase assembled with fVa^WT^ (Fig. 8). In addition, when prothrombin is activated by prothrombinase assembled with fV^ΔB9/Q3^, there is slight persistence (lingering) of meizothrombin throughout the activation process that is accompanied by a small delay in thrombin formation (Fig. 7*B*).

To understand the effect of the nine amino acid deletion on the ability of prothrombinase assembled with fV^ΔB9/Q3^ to cleave and activate prothrombin, we compared the rates of prothrombin consumption by prothrombinase assembled with fV^ΔB9/Q3^ to the rate of prothrombin consumption by prothrombinase assembled with fVa^RQQ^ that has a *k*_cat_ value comparable with prothrombinase assembled with the mutant cofactor and a *K_m_* value similar to prothrombinase assembled with fVa^WT^, using similar experimental conditions (60 nm fV^ΔB9/Q3^ and 80 nm fVa^RQQ^). The kinetic data are shown in Table 2, and the analysis of the time course is provided in Fig. 8 and shows that prothrombin consumption by prothrombinase assembled with fVa^RQQ^ is similar to the rate of prothrombinase assembled with fV^ΔB9/Q3^ (12.7 and 14 mol of prothrombin consumed per s/mol of factor Xa, respectively). This result is expected because the *k*_cat_ value of prothrombinase assembled with either fV^ΔB9/Q3^ (80 nm) or fVa^RQQ^ (60 nm) is similar (Table 2). The rate of prothrombin consumption by prothrombinase assembled with fVa^WT^ was 21 mol of prothrombin consumed per s/mol of factor Xa. These data demonstrate that prothrombinase assembled with fV^ΔB9/Q3^ has a similar capability to function as prothrombinase assembled with fVa^RQQ^, even though the specificity constant of the enzyme for prothrombin is substantially lower (Table 1).

To verify which cleavage in prothrombin is specifically affected by prothrombinase assembled with fV^ΔB9/Q3^, we used recombinant prothrombin molecules that have only one cleavage site for prothrombinase (at Arg^320^ for rMZ-II and at Arg^271^ for rP2-II, data not shown) (24). The data demonstrate a small difference between the rates of activation of rMZ-II by prothrombinase assembled with fV^ΔB9/Q3^ as compared with the rates of rMZ-II activation by prothrombinase made with fVa^WT^. No significant difference was observed when rP2-II was incubated with prothrombinase assembled with fV^ΔB9/Q3^ as compared with cleavage of rP2-II by prothrombinase assembled with fVa^WT^. These data confirm our findings obtained with plasma-derived prothrombin. Overall, the data demonstrate that the rate of prothrombinase-mediated cleavage at Arg^320^ in prothrombin is slightly affected by prothrombinase assembled with fV^ΔB9/Q3^.

To determine the effect of deleting nine amino acids from the B region of fV on the cleavage of prothrombin at Arg^271^ alone, we compared the rate of cleavage of FPR-meizothrombin by prothrombinase assembled with either fVa^WT^ or fV^ΔB9/Q3^ (data not shown). The data demonstrate a 1.8-fold delay for cleavage of FPR-meizothrombin at Arg^271^ by prothrombinase assembled with fV^ΔB9/Q3^ as compared with the same reaction catalyzed by prothrombinase assembled with fVa^WT^. Thus, the nine amino acid deletion in fV^ΔB9/Q3^ does not have a large inhibitory effect on prothrombinase cleavage at Arg^271^. Overall, these data strongly suggest that amino acid region 1000–1008 represents a crucial regulatory amino acid stretch because it covers the important binding site(s) of fVa for fXa. The large increase in the *K_m_* value of the enzymatic reaction using prothrombinase assembled with fV^ΔB9/Q3^ is most likely due to the steric hindrance by the bulky B region still attached to the heavy chain of the cofactor.

## DISCUSSION

**FIGURE 9.**
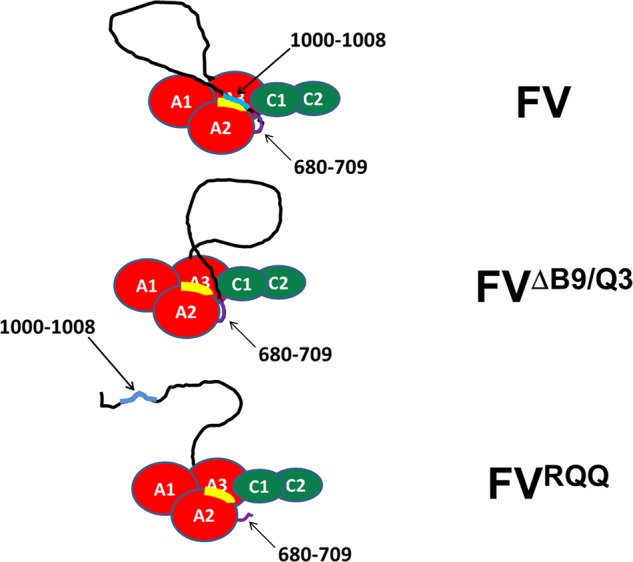
**Schematic interpretation of the data.** In full-length fV, the basic amino acid region 1000–1008 from the B domain (*blue*) covers the fXa-binding site(s) located on the A2 and A3 of the molecule (*yellow*). The acidic 680–709 region is shown in *purple*. From the kinetic and binding data obtained, we can hypothesize that fV^ΔB9/Q3^ and fVa^RQQ^ are able to have a productive interaction with prothrombin because both the fXa and prothrombin-bindings sites are available.

The data presented herein demonstrate for the first time that the basic amino acid region composed of residues 1000–1008 and located in the middle portion of the B domain of fV is a dynamic regulator for the binding of the active cofactor to fXa within prothrombinase. We have shown that three out of the six amino acid residues from the heavy chain of fVa that are involved in fXa binding are acidic in nature (17, 18, 22, 34, 51). Our previous work with recombinant fVa molecules containing specific point mutations of these amino acids demonstrates a significant decrease in affinity for fXa when the residues were mutated two by two and a dramatic decrease in affinity (50-fold) when four amino acids were mutated simultaneously (17, 18). It is thus logical to hypothesize that in the absence of injury to the endothelium these acidic amino acids interact with the 1000–1008 region from the B domain of fV to neutralize the unnecessary interaction of the pro-cofactor with fXa (Fig. 9). Upon vascular injury and thrombin formation, this region is removed following proteolytic cleavage. To the best of our knowledge, this is the first time that this part of the molecule alone has ever been functionally investigated with a full-length recombinant fV molecule.

Previous work using recombinant B domainless factor V suggested that two regulatory sequences of 45 and 44 amino acids each (963–1008 and 1492–1538, respectively) are required to keep the pro-cofactor in a quiescent state (14, 15, 49). The requirement for these two sequences was deduced from experiments comparing several B domainless molecules where large amino acid portions of the B domain were either added or deleted to a recombinant fV molecule lacking most of the B domain (680 amino acids) (14). Conclusions were based on results from experiments using clotting activities, direct binding assays, and primary thrombin generation plots using prethrombin-2 or prothrombin as substrates. No kinetic studies of the mutant molecules were provided (15, 49). In addition, although earlier data showed that amino acid region 963–1008 alone was required to keep the pro-cofactor in a quiescent state (15), more recently Bos and Camire (49) showed that, in addition to region 963–1008, amino acid region 1492–1538 was also necessary to keep the pro-cofactor in an inactive state. Moreover, a construct without sequence 1000–1008 (FV-956 (15)) was shown to have clotting activity comparable with a recombinant molecule harboring this biologically important stretch of amino acids (FV-B8-BR3 (49)). Results presented herein were obtained using minimally altered full-length recombinant fV, are in sharp contrast with those conclusions, provide a detailed kinetic and thermodynamic analysis of the results obtained with each full-length recombinant fV mutant, and identify a short amino acid stretch from the B domain of the pro-cofactor as the only amino acid sequence required to keep fV in a quiescent state. Our work using minimally modified full-length recombinant fV provides a new physiological role for this basic region. Specifically, amino acid region 1000–1008 has the ability to maintain the pro-cofactor in a latent state by impairing its interaction with fXa (Fig. 9).

Prothrombinase is an enzymatic complex composed of two subunits as follows: a catalytic subunit (fXa) and a regulatory subunit (fVa) assembled on a procoagulant membrane surface in the presence of divalent metal ions. The catalytic subunit alone can cleave and activate prothrombin; however, the rate of thrombin formation is slow and incompatible with survival. Addition of phospholipid vesicles increases the *V*_max_ of the reaction by a modest 7-fold, whereas the *K_m_* of fXa is increased by 380-fold (52). Addition of fVa to the phospholipid/fXa mixture has no significant effect on the *K_m_* value of the enzyme, but it increases the *V*_max_ of the enzymatic reaction by ∼3000-fold. Thus, the fXa-fVa interaction mostly affects the catalytic efficiency of the enzymatic reaction (*k*_cat_, turnover number) toward prothrombin and demonstrates that fVa is required for the efficient prothrombin to thrombin formation by fXa. As a consequence, fVa's contribution to prothrombinase once bound to fXa is most likely materialized by positioning prothrombin in an optimal orientation for efficient fXa cleavage and timely thrombin formation. We and others have repetitively demonstrated that the heavy chain of fVa, and in particular the COOH-terminal acidic region of the heavy chain of fVa, immediately adjacent to the cleavage site at Arg^709^, is required for optimal rates of prothrombin cleavage and thrombin formation (41, 42, 53). Our present data clearly demonstrate that amino acid sequence ^1000^KTRKKKKEK^1008^, containing a cluster of basic amino acids, appears to control spontaneous binding of fV to fXa. This stretch of amino acids being ionic in nature is most likely found on the surface of the full-length derivative, is exceedingly conserved among species (Fig. 2), providing further proof to its fundamental purpose in coagulation, and is in close proximity to a thrombin cleavage site.

We show that fV^ΔB9/Q3^ can act as a cofactor for the increase in prothrombinase activity as compared with fXa alone with respect to prothrombin cleavage, albeit with a decreased *k*_cat_ (Table 2). However, increasing the concentration of fV^ΔB9/Q3^ within the mixture results in an increased *k*_cat_ value (*V*_max_/*E_T_*) because of the increase in the number of productive collisions between prothrombinase assembled with fV^ΔB9/Q3^ and prothrombin. The increase in *k*_cat_ is not due to the increase in enzyme concentration, because the concentration of prothrombinase remains essentially constant over a large increase in fV^ΔB9/Q3^ concentration (15–80 nm, Table 2). However, increasing the concentration of fV^ΔB9/Q3^ also results in an increase in the *K_m_* value of the reaction. Thus, it is logical to hypothesize that most likely an excess concentration of the bulky B domain still attached to the end of the heavy and light chains of the cofactor impairs prothrombin interaction with the enzyme in a competitive manner with respect to prothrombin.

Removal of the nine basic amino acids from the B region had a highly significant effect on the dissociation constant of recombinant fV^ΔB9/Q3^ for fXa when compared with fV^Q3^. We have measured the *K_D_*_(app)_ of fV^ΔB9/Q3^ for factor Xa under physiological concentrations of fV (1–10 nm). Under these conditions, the *K_m_* value of prothrombinase assembled with fV^ΔB9/Q3^ is similar to the *K_m_* value of prothrombinase assembled with fVa^WT^ (Fig. 4 and Tables 1 and 2). Increasing the concentration of the mutant pro-cofactor within prothrombinase, while keeping fXa constant, resulted in increased *K_m_* and *k*_cat_ values of the enzyme (Table 2). As a consequence, even though the *K_m_* value of prothrombinase assembled with the mutant pro-cofactor was significantly increased in an *in vitro* assay utilizing a chromogenic substrate and much higher (saturating) supraphysiological concentrations of fV^ΔB9/Q3^ (60 nm), suggesting impaired interaction between prothrombinase assembled with high concentrations of fV^ΔB9/Q3^ and prothrombin, the clotting activity of fV^ΔB9/Q3^, which is a measure of its physiological activity *in vivo* (because clotting requires very little active cofactor (54, 55)), was indistinguishable from the clotting activities of fVa^WT^ or fVa^plasma^. These results were verified by performing similar experiments using high concentrations of fVa^RQQ^. Thus, under physiological conditions fV^ΔB9/Q3^ can promote efficient thrombin generation, which in turn will result in efficient fibrin clot formation. Because it has been well established that even minute concentrations of fVa (0.1%) in mice provide rescuing effects on physiology (56, 57), it therefore becomes obvious that even a small mutation (one amino acid) within the 1000–1008 region of the B domain could have profound and devastating clinical implications for normal hemostasis. This may be the reason that to date there are no documented occurrences of a mutation in this precise highly basic and conserved stretch of amino acids because any amino acid alteration in this region is incompatible with survival.

It is important to note that to test the overall function of the recombinant pro-cofactor, functional assays alone are insufficient in obtaining a definitive answer as to the exact function of a region of the pro-cofactor on prothrombinase function and need to be coupled with experiments employing SDS-gel electrophoresis that provide for a visual inspection of the activation pathways of prothrombin. We have observed an ∼250-fold decrease in the substrate specificity of prothrombinase assembled with fV^ΔB9/Q3^, in the presence of three times the physiological concentration of fVa, which was due mostly to its inability to interact with prothrombin. In contrast, using physiological concentrations of recombinant molecules, we show that fV^Q3^ had impaired clotting activity and did not show any interaction with fXa compared with the ability of fVa^plasma^, fVa^WT^, fVa^RQQ^, and recombinant fV^ΔB9/Q3^ molecules. Although fV^ΔB9/Q3^ had similar clotting activity as the activated wild type cofactor molecule, the recombinant protein is completely resistant to cleavage and activation by thrombin as seen with fV^Q3^. Altogether, these data validate our results obtained with fV^ΔB9/Q3^ and suggest that this peptide sequence composed of mostly basic amino acids is indispensable in preventing unwanted clot formation.

Activation studies of fV and the analysis of the function of the B domain are crucial because the penultimate step of the common pathway during blood clotting is the assembly of prothrombinase, which is necessary for thrombin formation. Our data provide a logical explanation for the sequential cleavage and activation of human fV by thrombin. Initial cleavage at Arg^709^ is necessary to expose both the COOH-terminal portion of fVa heavy chain necessary for optimal prothrombin interaction and the fXa-binding site (Fig. 9). Cleavages at Arg^1018^ and Arg^1545^ release additional constraints on the pro-cofactor that can then fully interact with fXa and efficiently produce thrombin. Results from this project provide a unique role for the B domain residues in fV involved in the regulation of its coagulant cofactor effects. Further experiments involving alanine scanning mutagenesis in full-length recombinant fV will provide concrete insights into the identity of the specific amino acids within the 1000–1008 region that inhibit fXa binding. This study provides significant new and original information of a commanding stretch of amino acids that could be used in creating safer alternative therapies for thrombotic and/or hemophilic patients by synthesizing molecules targeting a regulatory sequence within fV that is not currently in existence.

## Footnotes

3 The abbreviations used are:

fV: factor V
fV^WT^: wild type recombinant human fV
fVa^WT^: wild type recombinant human fV incubated with thrombin
fV^RQQ^: recombinant human fV with Arg^1018^ and Arg^1545^ mutated to glutamine
fVa^RQQ^: recombinant human fV with Arg^1018^ and Arg^1545^ mutated to glutamine following incubation with thrombin
fV^Q3^: recombinant human fV with the thrombin activation cleavage sites (Arg^709^/Arg^1018^/Arg^1545^) mutated to glutamine (fV^Q3^)
fV^ΔΒ9^: recombinant human fV with region 1000–1008 deleted (nine amino acids)
fVa^ΔΒ9^: recombinant human fV with region 1000–1008 deleted incubated with thrombin
fV^ΔΒ9/Q3^: recombinant human fV with region 1000–1008 deleted and the thrombin activation cleavage sites (Arg^709^/Arg^1018^/Arg^1545^) mutated to glutamine
DAPA: dansylarginine-*N*-(3-ethyl-1,5-pentanediyl)-amide
FPR: d-Phe-Pro-Arg.
